# Education level and misuse of antibiotics in the general population: a systematic review and dose–response meta-analysis

**DOI:** 10.1186/s13756-022-01063-5

**Published:** 2022-02-03

**Authors:** Narmeen Mallah, Nicola Orsini, Adolfo Figueiras, Bahi Takkouche

**Affiliations:** 1 Department of Global Public Health, Karolinska Institutet, Stockholm, Sweden; 2 Department of Preventive Medicine, University of Santiago de Compostela, R/ San Francisco, s/n, 15782 Santiago de Compostela, Spain; 3 Centro de Investigación Biomédica en Red de Epidemiología y Salud Pública (CIBER-ESP), Madrid, Spain; 4 Health Research Institute of Santiago de Compostela (IDIS), Santiago de Compostela, Spain

**Keywords:** Antibiotic, Dose–response, Education, Meta-analysis, Misuse

## Abstract

**Background:**

Numerous studies evaluated the association of education level with misuse of antibiotics by the general population, yet divergent findings were reported. Therefore, a meta-analysis was conducted to summarize this association.

**Methods:**

A categorical and continuous dose–response meta-analysis of the association of education level with antibiotic misuse was undertaken. Summary odds ratios (ORs) and their 95% confidence intervals (CIs) were estimated using random-effect model.

**Results:**

The meta-analysis included 85 studies from 42 countries of different socioeconomic status. Compared to low education (≤ 9 years), medium education (> 9–12 years) is associated with 20% lower odds of antibiotic misuse in high-income countries (OR = 0.80; 95% CI 0.66, 0.97), while high education (> 12 years) is associated with 14% lower odds of any aspect of antibiotic misuse (OR = 0.86; 95% CI 0.72, 1.03). The association is more pronounced in Middle East (OR = 0.64; 95% CI 0.42, 1.00) and countries of lower-middle economies (OR = 0.67, 95% CI 0.41, 1.11). Inversely, in Europe, high education is associated with 25% higher odds of antibiotic misuse (OR = 1.25, 95% CI 1.00, 1.58). Each additional year of education was associated with 4% lower odds of any aspect of antibiotic misuse in lower-middle economies (OR = 0.96; 95% CI 0.92, 1.00) and in Middle East (OR = 0.96; 95% CI 0.93, 1.00). Conversely, it was associated with 3% higher odds of antibiotic storage, a specific type of misuse (OR = 1.03, 95% CI 1.01, 1.06).

**Conclusion:**

Individuals misuse antibiotics irrespective of their education level. Intervention programs to enhance the proper use of antibiotics should target all communities independent of their education level.

**Supplementary Information:**

The online version contains supplementary material available at 10.1186/s13756-022-01063-5.

## Background

Antibiotic resistance continues to represent an essential public health problem despite efforts exerted worldwide to reduce the causes of the resistance. It affects all regions irrespective of the country socioeconomic status [1], and cause heavy clinical, social and economic burdens. More than 700,000 individuals die each year due to antibiotic resistant bacteria, and it is expected that the annual mortality rate from antibiotic resistance will exceed that of major diseases by 2050 [2]. Furthermore, projections show that the economic shortfalls due to this problem will increase and will soon be equivalent to those seen in the 2008–2009 global financial downturn [3].

Antibiotic resistance is exacerbated by excessive use of antibiotics in agriculture, food and feed chain, imprecise antibiotic prescription by physicians, and misuse of antibiotics by the patients. Individuals tend to misuse antibiotics by consuming these drugs without medical prescription (self-prescription) or by using them based on medical advice but without adherence to the physician’s instructions such as modifying the prescribed dose, truncating or prolonging the treatment duration or not taking the antibiotics on time [4].

The use of antibiotics without prescription is salient worldwide, with a pooled prevalence exceeding 75% in low- and middle-income countries [5], and reaching 66% in some regions of high-wealth countries such as the United States [6].

Previous reports also indicated that more than one-third of patients do not fully adhere to antibiotic treatment regimen [7], around 50% cease their antibiotic treatment upon improvement [8], and one-third store antibiotic leftover for future use [7].

Besides determinants of antibiotic misuse such as female gender, youth and old age, lack of access to healthcare facilities and easy access to antibiotics [6, 9], educational level was suggested to be associated with misuse. Yet inconsistent findings exist regarding this association. Several studies reported an association between low education and antibiotic misuse [10]. Inversely, several other studies reported that high education level is associated with greater risk of antibiotic misuse [11], while some studies failed to find any association [12] Accordingly, to summarize those findings, we aimed in the present study to carry out a systematic review and dose–response meta-analysis of the association of education level with antibiotic misuse.

## Methods

We registered this systematic review and meta-analysis in the PROSPERO database (Protocol ID: CRD42021233425) and carried it out according to PRISMA guidelines. The main outcome, antibiotic misuse in the general population, was defined as the occurrence of any of the following practices: unprescribed use of antibiotics (self-medication), non-adherence to treatment guidelines and storage of antibiotics leftover for future use.

### Literature search and study selection

We searched Medline, EMBASE, the five regional bibliographic databases of the World Health Organization (WHO), the Conference Proceedings Citation Index-Science, and the Open Access Theses and Dissertations until January 2021. In Medline, we used the following search term without any language, date or other restrictions: (Socioeconomic Factors OR education) AND (antibiotic*) AND ((compliance) OR (adherence) OR (Nonprescription Drugs / administration & dosage* [MeSH]) OR (misuse) OR (irrational use) OR (left-over)). We also ran the search using related free-text words. Then, we adapted the syntaxis to complete the search in the other databases. We manually checked the reference lists of included studies and those of relevant review reports to supplement the electronic search. The list of the examined review reports is provided in Additional file 3.

We included studies that (1) measured the association between education and any aspect of antibiotic misuse (i.e., unprescribed use of antibiotics for oneself or for another person, non-adherence to antibiotic treatment guidelines or storage of antibiotic leftover), (2) defined the measured level of education, (3) reported odds ratio (OR) or risk ratio (RR) and their 95% confidence intervals (CIs) or sufficient data for their calculation. We excluded from the meta-analysis studies that only compared students according to their university year.

### Data extraction and synthesis

We collected information on: (1) study source: author’s last name and publication year, (2) settings and participants’ demographic characteristics, (3) study design, (4) exposure: levels of education, (5) for each education level: ORs and its 95% CIs, total number of participants, and number of individuals who reported antibiotic misuse. We extracted the ORs adjusted for the largest number of variables, (6) restriction, adjustment, or matching variables, and (7) type of antibiotic misuse. In studies reporting more than one type of antibiotic misuse, we extracted the data of all types of misuse, and treated each type of misuse as a separate study unit in the dose–response analysis. We contacted the authors to inquire about the number of individuals who misused and those who did not misuse antibiotics per each education level, when needed.

In addition to data reported in the included studies, we obtained the classification of countries wellness (low, lower-middle, upper middle and high income) from the World Bank [13] and used for geographic distribution the classification by region of the World Health Organization (African, Eastern-Mediterranean, European, Region of the Americas, South-East Asia and Western Pacific) [14].

### Dose definition

We defined the term “dose” as the level of education in years. Education level classification varies between countries, hence, to standardize the education levels across studies of different parts of the world, we transformed it to years of education according to the education system used in each country. We set the dose as the midpoint of the upper and lower boundaries of each education level.

### Statistical analysis

We performed a dose–response meta-analysis using a one-stage mixed-effects model taking into account heterogeneity across studies [15]. We carried out categorical and continuous approaches.

#### Categorical approach

To facilitate tabular presentation of the summary ORs, and in line with other studies, we further recategorized education level into low (≤ 9 years), medium (> 9–12 years), and high (> 12 years) levels and used low education level as a referent.

#### Continuous approach

We applied a linear function to estimate a summary OR of antibiotic misuse associated with an increase of 1 year of education. Then, we flexibly modelled education using restricted cubic splines with 3 knots fixed at 10th, 50th and 90th percentiles of its distribution, and tested departure of the second spline from linearity.

We undertook stratified dose–response analyses of the level of education with antibiotic misuse. The analysis was stratified by study design, type of antibiotic misuse, geographic region, country wellness, methods of exposure ascertainment, comparability (adjustment for age and gender), and publication year, using 2015, the year of publication by the World Health Organization of the global action plan against antibiotic resistance, as a cut-off limit [16].

### Quality assessment

We appraised the quality of the studies included in the meta-analysis using the Newcastle–Ottawa Scale for cohort and cross-sectional studies [17, 18]. Two epidemiologists (AF and NM) performed the quality assessment, and disagreements were resolved by consensus through discussion with a third epidemiologist (BT). Seven criteria were evaluated. The following five criteria were common to cohort and cross-sectional designs: (1) justified sample size; (2) application of previously tested or validated questionnaire to ascertain education level; (3) use of external assessment in addition to questionnaire to ascertain antibiotic misuse; (4) described and appropriate statistical analysis; and (5) adjustment, matching or restriction for age and gender. Additionally, we evaluated two criteria that were specific to study design. For cohort studies we checked (1) if the study sample was representative of the general population and (2) if the response rate was more than 50%. For cross-sectional studies we examined (1) if the study population was defined; (2) if the response rate was reported. We gave one point for the fulfilment of each of the seven criteria, and then summed those points to obtain a quality score of a maximum of seven points. When the information on an item was absent in the publication, this item scored zero point.

### Publication bias

We checked publication bias visually using funnel plot, and formally through Egger’s test [19] and the trim and fill method [20].

## Results

### Literature search and study selection

**Fig. 1:**
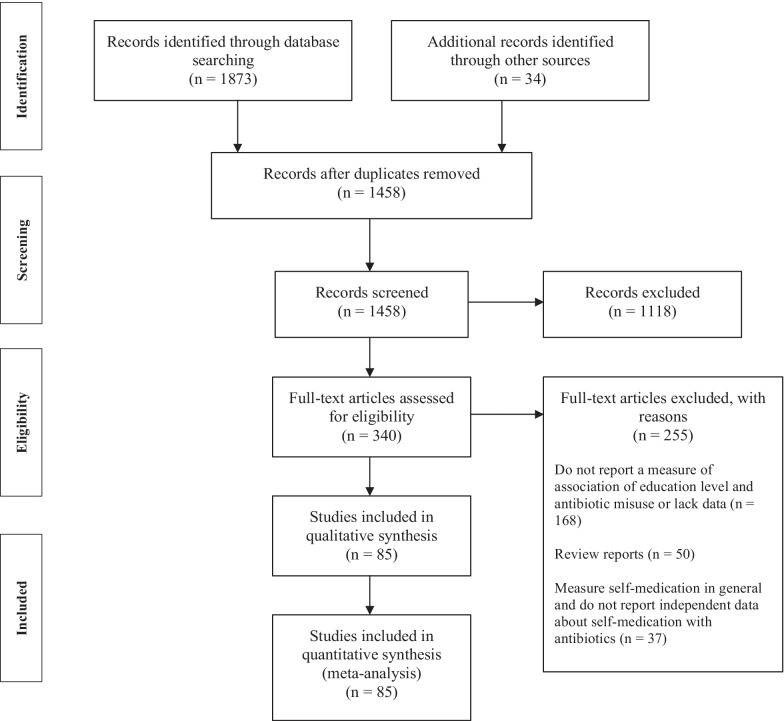
Flow diagram of the selection of studies on the association of education level and misuse of antibiotics

Out of 1458 identified studies, 85 fulfilled the inclusion criteria and were included in the meta-analysis (Fig. 1). The general characteristics of the included studies are summarized in Table 1 and Additional files 1 and 2 and their references are provided in Additional file 3. Out of all contacted authors to inquire about missing data in Table 1, three answered our inquiry [21–23]. Eighty-three studies were of cross-sectional design and the remaining two studies were cohort studies. They encompassed a total population of 85,789 subjects, out of whom 24,579 had misused antibiotics as follows: use without prescription (N = 15,780), storage of antibiotics (N = 6077), non-adherence to antibiotic treatment regimen (N = 2293) and several concomitant types of misuse (N = 429). When studies provided data for several types of misuse, each type was treated as a separate study, making a total of 94 studies introduced in the dose–response analysis. The studies were published between 2000 and 2021 and originated from 42 different countries. All studies were published in English, except five that were available in Croatian, Italian, and Spanish.

**Table 1:** General characteristics of studies about education level and misuse of antibiotics

Source	Country	Age (years)	Gender	Population	Type of misuse	Dose	N		n	OR (95% CI)	Adjustment, restriction or matching factors
*Cohort studies*											
Afari-Asiedu 2020	Ghana	Adults (< 20 to > 60)	M: 36.0% F: 64%	General population	Any misuse	0.0	283	27		1.00	Age, gender, marital status, occupation, place of residence, health insurance, type of drug supplier
						3.5	176	19		0.90 (0.40, 1.70)	
						8.0	138	22		0.60 (0.30, 1.10)	
						13.0	164	34		0.40 (0.20, 0.70)	
Ho 2010	Australia	Adults (≥ 18) ≤ 30 to > 45	M: 43% F: 57%	Emergency department attendants	Non-adherence	3.0	16	4		1.00	Age, gender, marital status, employment status, having a regular general practitioner, number of usual medications, place of birth, use of herbal medicine
						9.0	70	16		0.79 (0.46, 4.55)	
						12.0	35	10		0.39 (0.06, 2.78)	
						15.5	71	9		0.33 (0.08, 1.43)	
*Cross-sectional studies*											
Moktan 2021	India	Adults (> 18)	M: 61.3% F: 38.7%	Attendants of primary care centers	Use without prescription	0.0	28	12		1.00	Age, gender, marital status, income, public and private clinics, frequency of doctors’ consultation, family/friend influence (other family members self-medicating with antibiotics), symptoms (minor illness)
						6.5	206	65		0.61 (0.28, 1.37)	
						14.5	158	59		0.79 (0.35, 1.80)	
						19.0	112	35		0.61 (0.26, 1.42)	
Bianco 2020	Italy	Adults mean ± SD: 47.8 ± 16.7	M: 41.2% F: 58.8%	Attendants of primary care centers	Use without prescription	4.0	–	–		1.00	Age, gender, marital status, employment status, nationality, having a kid who had used antibiotics in the last year
						11.0	–	–		1.38 (0.86, 2.21)	
						16.0	–	–		1.34 (0.78, 2.29)	
Elmahi 2020	Sudan	Adults (> 18)	M: 52.8% F: 47.2%	General population	Use without prescription	6.0	35	23		1.00	Age
						14.5	211	125		0.76 (0.36, 1.61)	
Hallit 2020	Lebanon	Adults mean ± SD: *bad practice* 33.33 ± 8.80 *good practice* 31.49 ± 6.68	M: 48.5% F: 51.5%	Children caregivers	Any misuse	3.0	48	40		1.00	Age
						11.0	40	22		0.24 (0.09, 0.65)	
						14.5	116	41		0.11 (0.05, 0.26)	
Mallah 2020	Lebanon	Adults (> 18)	M: 19.4% F: 76.8%	Children caregivers	Any misuse	6.0	252	31		1.00	Age, gender, income, area of residence, access to medical care facilities, frequency of telephone medical consultation
						14.5	1157	62		0.40 (0.27, 0.64)	
Nusair 2020	Jordan	Unrestricted age group > 60	M: 36.6% F: 63.4%	General population	Use without prescription	0.0	51	18		1.00	–
						5.5	52	20		1.15 (0.51, 2.55)	
						11.5	273	104		1.13 (0.60, 2.11)	
						15.5	1505	632		1.33 (0.74, 2.38)	
Rathish 2020	Sri Lanka	Unrestricted age group Mean ± SD: 36.0 ± 21.0	M: 47% F: 53%	General population	Use without prescription	11.0	252	247		1.00	–
						13.5	132	127		3.95 (0.74, 21.10)	
Shah 2020	Pakistan	≥ 15	M: 33% F: 67%	General population	Use without prescription	0.0	45	25		1.00	Age
						4.5	231	30		0.12 (0.06, 0.24)	
						10.5	198	54		0.30 (0.15, 0.58)	
						14.5	221	71		0.38 (0.20, 0.73)	
Xu 2020	China	Parents with children < 13 years old	M: 21.5% F: 78.5%	Children caregivers	Use without prescription	4.5	1344	–		1.00	Age, gender, income, medical background, residential location
						11.0	1771	–		0.85 (0.58, 1.25)	
						14.5	3164	–		0.77 (0.53, 1.14)	
					Antibiotic Storage	4.5	1344	–		1.00	
						11.0	1771	–		1.38 (1.19, 1.60)	
						14.5	3164	–		1.55 (1.33, 1.81)	
Ateshim 2019	Eritrea	Median (IQR): 37 (24)	M: 41.2% F: 58.8%	General population	Use without prescription	0.0	32	11		1.00	Age, gender, income, marital status, occupational status, knowledge about antibiotics, attitudes towards antibiotics
						3.0	64	22		1.96 (0.56, 6.88)	
						6.5	95	33		1.62 (0.46, 5.72)	
						9.5	246	111		1.92 (0.55, 6.69)	
						14.0	140	81		2.8 (0.74, 10.63)	
Bogale 2019	Ethiopia	18 to > 60	M: 41.3% F: 58.7%	General population	Use without prescription	0.0	–	26		6.39 (1.45, 28.19)	Age, gender, income, marital status, residential location, occupational status, healthcare profession
						4.5	–	72		1.50 (0.82, 2.74)	
						10.5	–	75		1.46 (0.74, 2.87)	
						14.5		81		1.00	
Bulabula 2019	South Africa	Mean ± SD: 29 ± 6.1	F: 100%	Pregnant women attending public hospital	Use without prescription	5.0	14	–		1.00	Age, gender, income, residential location,, knowledge about antibiotics, attitudes towards antibiotics
						11.0	228	–		1.25 (0.15, 10.10)	
						15.5	5	–		1.60 (1.03, 2.55)	
Ekambi 2019	Cameroon	≥ 15 Mean ± SD: 35.02 ± 10.5	M: 52% F: 48%	Attendants of pharmacies	Use without prescription	3.0	25	8		1.00	Age, gender, marital status, occupation
						10.0	86	28		1.02 (0.94, 2.66)	
						15.0	66	33		2.07 (1.10, 4.00)	
Mate 2019	Mozambique	Adults (> 18) Median (IQR): 33 (25–47)	M: 26.9% F: 73.1%	General population	Use without prescription	0.0	69	17		1.00	Age
						4.0	344	63		0.69 (0.37, 1.26)	
						11.5	555	119		0.83 (0.47, 1.50)	
						14.5	114	28		1.00 (0.50, 1.99)	
					Non-adherence	0.0	65	15		1.00	
						4.0	334	88		1.19 (0.64, 2.23)	
						11.5	531	177		1.67 (0.91, 3.05)	
						14.5	110	33		1.43 (0.70, 2.90)	
Mukattash 2019	Jordan	20 to ≥ 50	M: 15.8% F: 84.2%	Children caregivers	Use without prescription	6.0	205	106		1.00	Age
						18.0	1487	554		0.56 (0.42, 0.75)	
Rajendran 2019	India	Adults (> 18)	M: 51% F: 49%	General population	Use without prescription	9.5	151	2		1.00	Age
						11.0	60	2		2.57 (0.35, 18.67)	
						14.0	540	21		3.01 (0.70, 13.00)	
Sun 2019	China	Parents with children < 13 years old	M: 23.5% F: 76.5%	Children caregivers	Antibiotic storage	5.0	2519	1026		1.00	Age, gender of the parents, gender of the child, socioeconomic characteristics (residential location and GDP per capita), health insurance, specialty (medical vs non-medical)
						11.0	2765	1365		1.34 (1.20, 1.51)	
						14.5	4242	2189		1.50 (1.33, 1.70)	
Voidăzan 2019	Romania	Adults (20–80) Mean ± SD: 45 ± 12.5	M: 36.5% F: 63.5%	Attendants of primary care centers	Use without prescription	2.5	200	11		1.00	–
						9.0	564	94		3.43 (1.80, 6.56)	
						15.0	438	102		5.22 (2.73, 9.96)	
						22.5	790	77		1.86 (0.97, 3.56)	
Adisa 2018	Nigeria	Mothers of children under five	F: 100%	Mothers of children under five attending primary care centers	Use without prescription	3.0	19	8		1.00	Gender
						9.5	148	89		2.07 (0.79, 5.46)	
						14.5	160	73		1.15 (0.44, 3.02)	
Al-Qahtani 2018	Saudi Arabia	Adults (≥ 18)	M: 43.4% F: 56.6%	Attendants to primary care centers	Use without prescription	0.0	13	4		1.00	Age, gender
						3.5	39	13		1.13 (0.29, 4.35)	
						10.5	108	39		1.27 (0.37, 4.40)	
						15.5	281	119		1.65 (0.50, 5.49)	
						21.0	48	23		2.07 (0.56, 7.65)	
Chang 2018	China	Caregivers of children under seven	M: 33.3% F: 66.7%	Caregivers of children under seven	Use without prescription	4.5	336	–		1.00	Gender, child gender and age, number of children, health insurance, residential location
						11.0	1428	–		0.75 (0.57, 0.98)	
						14.5	1407	–		0.82 (0.62, 1.08)	
						19.0	187	–		0.86 (0.56, 1.32)	
Cheng 2018	China	30 to ≥ 71	M: 41.4% F: 68.3%	General rural population	Use without prescription	0.0	135*	83*		1.00	Age, gender, household size, health insurance
						3.5	165*	112*		1.19 (0.69, 2.05)	
						8.0	227*	139*		0.89 (0.50, 1.57)	
						13.0	97*	57*		0.84 (0.40, 1.74)	
Dar-Odeh 2018	Saudi Arabia	15–64 Mean ± SD: 29.08 ± 9.32	M: 40.7% F: 59.3%	Attendants of primary care centers	Use without prescription	6.0	138	47		1.00	Age
						15.5	333	81		0.63 (0.41, 0.97)	
						21.0	29	7		0.62 (0.25, 1.56)	
El-Sherbiny 2018	Egypt	Adults (≥ 18)	M: 50.3% F: 49.7%	Attendants of primary care centers	Any misuse	6.0	419	–		1.00	Age, gender, income, occupation, residential location
						14.5	181	–		0.52 (0.34, 0.79)	
Horumpende 2018	Tanzania	Median (IQR): 23 (20.5–36.5)	M: 53.33% F: 46.67%	General population	Use without prescription	0.0	17	9		1.00	Age, gender, income, marital status, occupation
						4.0	111	70		1.45 (0.46, 4.51)	
						10.0	172	95		1.02 (0.32, 3.25)	
Kamata 2018	Japan	20–69	M: 51.2% F: 48.8%	General population	Antibiotic storage	5.0	111	18		1.00	Age
						11.0	1265	115		0.52 (0.30, 0.89)	
						14.5	1932	254		0.78 (0.46, 1.32)	
					Non-adherence	5.0	111	26		1.00	
						11.0	1265	282		0.94 (0.59, 1.48)	
						14.5	1932	479		1.08 (0.69, 1.69)	
Ngu 2018	Cameroon	≥ 21 Median (IQR): 35 (27—49)	M: 44.8% F: 55.2%	Attendants of primary care centers	Use without prescription	0.0	29	14		1.00	Age
						6.5	279	115		0.75 (0.35, 1.62)	
Redzick 2018	Croatia	–	M: 26.1% F: 73.9%	Attendants of primary care centers	Use without prescription	4.5	32	8		1.00	Age
						10.5	312	38		0.42 (0.17, 0.99)	
						14.5	60	4		0.21 (0.06, 0.78)	
						19.0	140	12		0.28 (0.10, 0.76)	
Tong 2018	China	< 45 to > 60	M: 47.6% F: 52.4%	Attendants of primary care centers	Non-adherence	6.5	323	286		1.00	Age, gender, income, residential location, occupation, employment status, knowledge about antibiotics
						14.5	391	335		0.77 (0.50, 1.21)	
Abdelrahman 2017	Saudi Arabia	< 18 to > 65	M: 71.5% F: 28.5%	General population	Use without prescription	0.0	8	4		1.00	Age
						5.0	58	21		0.57 (0.13, 2.51)	
						11.0	360	117		0.48 (0.12, 1.96)	
						15.5	602	248		0.70 (0.17, 2.83)	
Albawani 2017	Yemen	≥ 18 Mean ± SD: 28.6 ± 7.7	M: 56.2% F: 43.8%	Attendants of pharmacies	Use without prescription	5.0	40	36		1.00	Age
						11.0	71	68		2.52 (0.53, 11.87)	
						15.0	229	191		0.56 (0.19, 1.66)	
Akici 2017	Turkey	≥ 15	M: 42.6% F: 57.4%	Attendants of primary care centers	Use without prescription	0.0	18	8		1.00	–
						4.5	18	6		0.63 (0.16, 2.41)	
						10.5	18	8		1.11 (0.29, 4.20)	
						14.5	18	9		1.25 (0.34, 4.64)	
					Non-adherence	0.0	9	6		1.00	
						4.5	9	6		1.00 (0.14, 7.10)	
						10.5	9	7		1.75 (0.22, 14.22)	
						14.5	9	7		1.75 (0.22, 14.22)	
					Antibiotic storage	0.0	8	3		1	
						4.5	9	4		1.67 (0.23, 12.22)	
						10.5	8	5		2.78 (0.37, 21.03)	
						14.5	9	6		3.33 (0.45, 24.44)	
Barber 2017	Philippines	Adults (≥ 18) Median (IQR): 32 (20)	M: 37.2% F: 56.7%	General population	Use without prescription	3.5	83	69		1.00	Age, gender, household size
						10.5	186	143		0.77 (0.30, 1.67)	
Erku 2017	Ethiopia	< 29 to > 60 Mean ± SD: 33.19 ± 10.82	M: 25.9% F: 74.9%	General population	Use without prescription	0.0	182	125		5.01 (2.62, 9.34)	Age, gender, income, marital status, household size, employment status, frequency of visiting health care institutions, satisfaction about healthcare service
						4.5	201	180		2.81 (1.32, 6.15)	
						10.5	179	159		1.96 (0.91, 4.51)	
						14.5	88	71		1.00	
Gebrekirstos 2017	Ethiopia	Adults (≥ 18) Median (IQR): 30 (16)	M: 60.6% F: 39.4%	Attendants of pharmacies	Use without prescription	0.0	152	75		1.00	Age, gender, income, marital status, employment status, household size, residential location, type of illness, healthcare insurance, previous experience with antibiotics, access to healthcare
						4.5	149	71		0.93 (0.59, 1.47)	
						10.5	260	121		0.89 (0.60, 1.33)	
						14.5	219	100		0.86 (0.60, 1.31)	
Hassali 2017	Malaysia	Adults (≥ 18) Mean ± SD: 28.7 ± 7.4	M: 42.75% F: 57.25%	General population	Any misuse	8.5	61	20		1.00	Age, gender, income, marital status, race, healthcare related occupation, employment status, health insurance
						14.0	339	111		0.83 (0.53, 1.31)	
Jamhour 2017	Lebanon	Adults (≥ 18)	M: 45.5% F: 54.5%	General population	Use without prescription	4.5	34	14		1.00	Age, gender, educational level, specialty (unrelated to health care)
						11.0	151	76		1.45 (0.68, 3.08)	
Kajeguka 2017	Tanzania	Adults (≥ 18) Mean ± SD: 35.4 ± 13.4	M: 48.0% F: 52.0%	General population	Use without prescription	0.0	26	16		1.00	Age, gender, income, marital status, employment status, self-treated condition
						4.0	87	33		0.38 (0.12, 0.94)	
						11.0	74	46		1.03 (0.41, 2.57)	
						16.0	133	72		1.10 (0.46, 2.64)	
Kurniawan 2017	Indonesia	Adults (≥ 18) Median (IQR): 45 (18–49)	M: 34.3% F: 65.8%	Attendants of primary care centers	Use without prescription	3.5	26	24		1.00	Age, gender, income, marital status, employment status, health insurance
						8.0	44	37		0.44 (0.08, 2.30)	
						11.0	139	101		0.22 (0.05, 0.98)	
						14.5	31	18		0.12 (0.02, 0.58)	
Senadheera 2017	Sri Lanka	Adults (≥ 18)	M: 31.30% F: 68.70%	General population	Use without prescription	11.0	362	14		1.00	Age, gender, income, employment status, health insurance, household size, receiving medical treatment in the last three months, knowledge of antibiotic name
						13.5	245	37		0.32 (0.17, 0.63)	
Torres 2017	Ecuador	Adults (≥ 18) range: 18—64	M: 45.6% F: 54.4%	General population	Use without prescription	3.5	51	22		1.00	Age
						10.5	173	83		1.22 (0.65, 2.28)	
						14.5	195	102		1.45 (0.78, 2.69)	
Abdulraheem 2016	Nigeria	Adults (≥ 18) Median (range): 25 (19–68)	M: 61.1% F: 38.9%	Attendants of primary care centers	Use without prescription	3.5	623	–		1.00	Age, gender, symptoms, occupation
						9.5	390	–		1.24 (1.13, 1.87)	
						14.5	137	–		1.32 (1.18, 1.96)	
Aleem 2016	Saudi Arabia	< 25 to ≥ 55	M: 39.5% F: 60.5%	Children caregivers	Use without prescription	6.0	102	29		1.00	Age, gender, income, household size
						15.0	508	42		0.23 (0.13, 0.39)	
Al Rasheed 2016	Saudi Arabia	Adults (> 18)	M: 23.2% F: 76.8%	Attendants of primary care centers	Use without prescription	0.0	100	13		1.00	Age, gender, marital status, employment status, occupation, symptoms
						3.5	186	16		1.59 (0.73, 3.45)	
						10.5	145	13		1.52 (0.67, 3.43)	
						15.5	145	50		0.28 (0.14, 0.59)	
Bilal 2016	Pakistan	Mean ± SD: 48.6 ± 4.4	M: 65.8% F: 34.2%	Attendants of primary care centers	Use without prescription	0.0	161	159		1.00	Age, residential location, specialty (non-medical related participants)
						9.5	86	81		0.20 (0.04, 1.07)	
						11.5	65	49		0.04 (0.01, 0.17)	
						14.5	50	26		0.01 (0.003, 0.06)	
						19.5	38	10		0.004 (0.001, 0.02)	
Nigigi 2016	Kenya	Adults (≥ 18)	M: 32.0% F: 68.0%	Attendants of primary care centers	Use without prescription	0.0	48	12		1.00	–
						4.0	115	35		1.13 (0.90, 1.97)	
						9.5	179	61		1.31 (0.92, 2.40)	
						13.5	20	9		1.57 (1.20, 2.50)	
Ding 2015	China	Adults ≤ 29 to > 50	M: 9.7% F: 90.3%	Children caregivers	Non-adherence	3.0	198	68		1.00	Age, access to healthcare (number of clinics)
						8.0	304	87		0.77 (0.52, 1.13)	
						13.0	120	47		1.23 (0.77, 1.97)	
Gebeyehu 2015	Ethiopia	Mean ± SD: *urban:* 34.1 ± 12.9 *rural:* 34.5 ± 11.5	M: 24.3% F: 75.7%	General population	Use without prescription	0.0	137	56		4.21 (1.47, 12.07)	Age, gender, educational level, marital status, employment status, residential location, household size, level of healthcare service satisfaction, knowledge on antibiotics use
						4.5	145	40		2.01 (0.93, 4.34)	
						10.5	89	14		1.01 (0.34, 2.94)	
						14.5	35	10		1	
Kusturica 2015	Serbia	Adults (≥ 18)	M: 20.1% F: 79.9%	General population	Antibiotic storage	4.5	27	9		1.00	Age
						10.5	169	75		1.60 (0.68, 3.76)	
						14.0	25	10		2.53 (0.89, 7.23)	
						16.5	162	84		2.15 (0.91, 5.08)	
Pavyde 2015	Lithuania	Adults (≥ 18) Mean ± SD: 38.6 ± 13.9	M: 42.1% F: 57.9%	Attendants of pharmacies	Use without prescription	6.0	298	–		1.00	Age, gender, residential location, parenthood, knowledge of antibiotics, occupation
						14.5	707	–		1.39 (0.90, 2.16)	
Yousif 2015	Saudi Arabia	Adults (≥ 18)	M: 57.0% F: 43.0%	General population	Use without prescription	6.0	96	80		1.00	Age, gender, income, marital status, employment status, residential location
						15.5	295	235		1.25 (0.71, 2.50)	
Cheaito 2014	Lebanon	Adults (≥ 18) Mean ± SD: 38.24 ± 13.7	M: 44.8% F: 55.2%	Attendants of pharmacies	Use without prescription	3.0	67	34		1.00	Age, gender, income, marital status, employment status, health insurance, having a reference doctor and frequency of consultation
						10.5	96	42		0.75 (0.40, 1.41)	
						17.5	156	58		0.57 (0.32, 1.02)	
Vásquez 2014	Ecuador	≥ 65	M: 45.8% F: 54.2%	General population	Use without prescription	3.0	463	52		1.00	Age
						11.5	205	26		1.15 (0.69, 1.90)	
Hu 2014	Australia	≥ 14 Mean ± SD: 33 ± 8.2	M: 45.0% F: 55.0%	General population	Use without prescription	6.0	67	14		1.00	Age, gender, income, residential location, employment status, marital status, parental status, language proficiency, main language spoken at home, health insurance
						14.5	402	134		1.89 (1.01, 3.53)	
Mihretie 2014	Ethiopia	≥ 17 Mean ± SD: 37.8 ± 12.2	M: 4.4% F: 95.6%	General population	Use without prescription	4.0	29	27		1.00	Age
						10.5	7	3		0.06 (0.01, 0.44)	
						14.5	9	4		0.06 (0.01, 0.42)	
Ramalhinho 2014	Portugal	Adults (≥ 18)	M: 48.7% F: 51.3%	General population	Use without prescription	4.5	308*	268*		0.84 (1.41, 11.78)	Age, gender, marital status, employment status, residential location, access to healthcare, chronic disease, easy access to unprescribed antibiotics, occupation
						11.0	445*	340*		1.25 (0.66, 5.45)	
						15.5	293*	223*		1.00	
Yu 2014	China	Adults ≤ 20 to > 40	M: 25.6% F: 74.4%	Children caregivers	Use without prescription	3.5	16	–		0.19 (0.05, 0.75)	Age, gender, child age, number of children, medical insurance
						8.0	371	–		1.07 (0.64, 1.81)	
						11.0	258	–		1.01 (0.62, 1.65)	
						14.5	203	–		1.00	
Abobotain 2013	Saudi Arabia	< 25 to ≥ 55	M: 39.5% F: 60.5%	Children caregivers	Use without prescription	6.0	102	29		1.00	Age, income, marital status, household size, number of children, healthcare related profession
						14.5	508	42		0.23 (0.13, 0.39)	
Ecker 2013	Peru	Mean ± SD: 31.4 ± 10.8	F: > 94.9%	Children caregivers	Use without prescription	3.5	188	–		1.00	Age, gender, caregiver-child relatedness, age of the child, access to healthcare
						9.0	781	–		1.2 (0.7, 1.9)	
						14.5	232	–		1.0 (0.5, 1.7)	
Ekwochi 2013	Nigeria	–	M: 59.0% F: 41.0%	Children caregivers	Use without prescription	3.0	31	19		1.00	–
						9.5	83	45		0.75 (0.32, 1.74)	
						14.5	96	34		0.35 (0.15, 0.80)	
Ivanovska 2013	Macedonia	Adults (≥ 18)	M: 31.3% F: 68.7%	General population	Use without prescription	0.0	11	4		1.00	Age, occupation
						5.0	41	21		1.83 (0.47, 7.25)	
						11.5	256	112		1.36 (0.39, 4.77)	
						16.0	94	37		1.14 (0.31, 4.15)	
					Antibiotic storage	0.0	11	6		1.00	
						5.0	41	28		1.79 (0.46, 6.97)	
						11.5	256	187		2.26 (0.67, 7.64)	
						16.0	94	66		1.96 (0.55, 6.97)	
Khalil 2013	Saudi Arabia	≥ 15	M: 80.8% F: 19.1%	Attendants of primary care centers	Use without prescription	4.5	107	93		1.00	–
						11.0	435	350		0.62 (0.34, 1.14)	
						15.5	233	180		0.51 (0.27, 0.97)	
						21.0	18	14		0.53 (0.15, 1.83)	
Moradi 2013	Iran	Mean ± SD: 29.2 ± 8.05	M: 33.3% F: 66.7%	Attendants of pharmacies and primary care centers	Non-adherence	2.5	23	16		1.00	–
						11.0	50	36		1.13 (0.38, 3.32)	
						15.5	19	6		0.20 (0.05, 0.75)	
Chan 2012	Hong Kong, China	Adults (≥ 18)	M: 46.0% F: 54.0%	General population	Non-adherence	3.0	47	–		1.00	Age, gender, antibiotic knowledge
						9.5	165	–		1.77 (0.76, 4.11)	
						14.5	150	–		2.18 (0.85, 5.55)	
Elberry 2012	Saudi Arabia	≥ 20	F: 100%	Mothers	Use without prescription	6.0	34	2		1.00	Gender
						15.5	116	11		1.68 (0.35, 7.96)	
						21.0	49	6		2.23 (0.42, 11.79)	
Grosso 2012	Italy	< 25 to > 65	M: 43.1% F: 56.9%	Attendants of primary care centers	Use without prescription	4.0	489	82		0.39 (0.26, 0.61)	Age, gender, occupation
						11.0	268	61		0.41 (0.26, 0.64)	
						16.0	176	74		1.00	
					Non-adherence	4.0	502	169		1.77 (1.13, 2.75)	
						11.0	276	45		0.79 (0.48, 1.31)	
						16.0	178	33		1.00	
Clark 2011	Azerbaijan	Adults (≥ 18)	M: 43.6% F: 56.4%	General population	Use without prescription	0.0	35	8		1.00	Age, gender, ethnicity, household size
						7.0	646	123		1.23 (0.45, 3.36)	
						10.5	74	14		1.25 (0.37, 4.17)	
						13.5	37	9		1.48 (0.37, 5.93)	
Widayati 2011	Indonesia	Adults (≥ 18)	M: 38.3% F: 61.7%	General population	Use without prescription	3.5	8	5		1.00	Age, gender, income, marital status, household size, employment status, healthcare insurance
						8.0	8	3		0.36 (0.05, 2.73)	
						11.0	31	17		0.73 (0.15, 3.60)	
						14.5	21	8		0.37 (0.07, 1.98)	
Barah 2010	Syria	Adults (≥ 18)	M: 52% F: 48%	General population	Use without prescription	5.0	80	54		1.00	Age
						11.0	83	66		1.87 (0.92, 3.80)	
						15.5	89	55		0.78 (0.42, 1.47)	
						21.0	90	57		0.83 (0.44, 1.57)	
Landers 2010	United States	Mean ± SD: 33.7 ± 8.7	F: 100%	General population	Use without prescription	4.5	31	11		1.00	Age, gender
						13.0	42	18		1.36 (0.52, 3.55)	
Sawalha 2010	Palestine	–	NA	General population	Antibiotic Storage	3.5	239	86		1.00	–
						10.0	591	162		0.67 (0.49, 0.93)	
Togoobaatar 2010	Mongolia	Mean ± SD: 35.4 ± 11.9	F: 100%	General population	Use without prescription	5.0	183	–		1.20 (0.70, 2.20)	Gender, occupation
						12.0	320	–		1.00	
Abasaeed 2009	United Arab Emirates	Adults ≤ 20 to > 50	M: 65.8% F: 34.2%	Attendants of a book fair	Antibiotic storage	3.5	163	37		1.00	Age
						11.0	191	48		1.14 (0.70, 1.87)	
						15.5	496	166		1.71 (1.14, 2.58)	
Ilhan 2009	Turkey	Adults (≥ 18) Mean ± SD: 39.5 ± 15.2	M: 38.7% F: 61.3%	Attendants of primary care centers	Use without prescription	4.0	906	141		1.00	Age, gender, income, marital status, employment status, household size, healthcare insurance (social security), perceived health status, presence of chronic diseases
						12.5	1787	373		1.43 (1.15, 1.78)	
Sawair 2009	Jordan	16–65	M: 46.1% F: 53.9%	Attendants of primary care centers	Use without prescription	0.0	14	7		1.00	Age, gender, income, marital status, employment status, healthcare insurance, smoking habits, self-reported health status, chronic comorbidities
						5.5	37	15		0.68 (0.20, 2.35)	
						11.5	203	77		0.61 (0.21, 1.81)	
						15.5	198	85		0.75 (0.25, 2.23)	
						21.0	25	10		0.67 (0.18, 2.49)	
Hadi 2008	Indonesia	Adults (≥ 18)	M: 37.4% F: 62.6%	Attendants of primary care centers	Use without prescription	2.5	63	26		1.00	Age, gender, income, residential location, ethnicity, household size, healthcare insurance
						6.0	402	192		1.30 (0.76, 2.22)	
Mistretta 2008	Italy	≥ 10 Mean ± SD: 53.7 ± 17.7	M: 41.3% F: 58.7%	Attendants of primary care centers	Use without prescription	2.5	144	124		1.00	Age, gender, occupation, family size
						7.0	165	142		0.73 (0.36, 1.46)	
						11.0	195	160		0.62 (0.30, 1.28)	
						16.0	132	86		1.37 (0.65, 2.86)	
Al-Azzam 2007	Jordan	Unrestricted age category > 17 to < 60	M: 48.8% F: 51.2%	General population	Use without prescription	0.0	315	86		1.00	–
						5.5	396	143		1.51 (1.09, 2.08)	
						11.5	709	313		2.10 (1.58, 2.81)	
						15.5	713	300		1.93 (1.45, 2.58)	
Grigoryan 2006	19 European countries	Adults (≥ 18)	M: 46.8% F: 53.2%	General population	Use without prescription	6.0	–	–		1.00	Age, gender, country, chronic diseases
						14.0	–	–		1.36 (1.10, 1.68)	
					Antibiotic storage	6.0	–	–		1.00	
						14.0	–	–		1.69 (1.47, 1.94)	
Awad 2005	Sudan	≤ 20 to > 60	M: 45.1% F: 54.9%	General population	Use without prescription	0.0	156	63		1.00	Age, gender, income
						3.5	285	155		1.76 (1.18, 2.61)	
						7.5	392	262		2.98 (2.03, 4.36)	
						10.0	474	413		9.99 (6.58, 15.18)	
						14.5	443	400		13.73 (8.77, 21.51)	
Parimi 2002	Trinidad and Tobago	Adults (≥ 18)	M: 33.3% F: 66.7%	General population	Use without prescription	3.5	126	34		1.00	Age
						9.5	323	71		0.76 (0.47, 1.22)	
						14.5	147	25		0.55 (0.31, 0.99)	
					Antibiotic storage	3.5	124	26		1.00	
						9.5	308	74		1.19 (0.72, 1.98)	
						14.5	147	28		0.89 (0.49, 1.61)	
Bi 2000	China	–	F: 100%	Children caregivers	Use without prescription	3.5	140	65		1.00	Gender
						8.0	442	259		1.63 (1.11, 2.39)	
						11.0	780	478		1.83 (1.27, 2.62)	
						14.5	97	65		2.34 (1.37, 4.01)	
Saradamma 2000	India	> 6	M: 48.5% F: 51.5%	General population	Use without prescription	4.0	87	38		1.00	–
						10.5	136	24		0.28 (0.15, 0.51)	
						14.5	137	8		0.08 (0.04, 0.18)	
						19.0	45	3		0.09 (0.03, 0.32)	

CI, confidence interval; F, female; IQR, interquartile range; M, male; n, number of participants who misused antibiotics; N, total number of participants per education level; OR, odds ratio; SD, standard deviation; *, data provided by author; –, not reported

### Education level and antibiotic misuse

#### Overall association

**Fig. 2:**
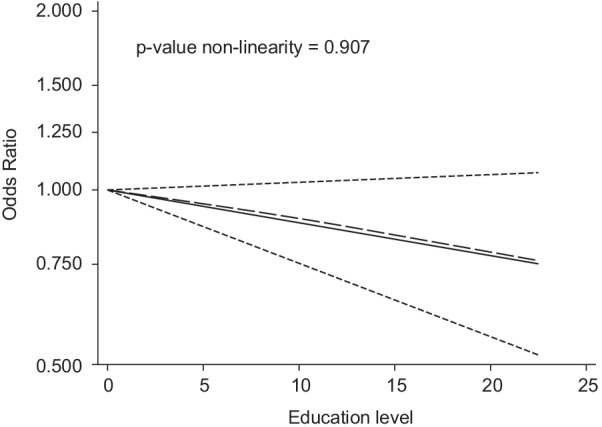
Trend of the association of education level with antibiotic misuse. Solid line represents the linear trend. Short-dashed lines represents 95% confidence intervals. Long-dashed line represents the non-linear restricted cubic spline approach

Compared to low education level (≤ 9 years), overall, medium education (> 9–12 years) is not associated with antibiotic misuse (OR = 0.94; 95% CI 0.83, 1.06) (Table 2). Nonetheless, high education level (> 12 years) is associated with 14% lower odds of antibiotic misuse, albeit with large confidence intervals (OR = 0.86; 95% CI 0.72, 1.03) (Table 2). In the continuous approach, the data accords well with a flat linear association between education and antibiotic misuse (OR = 0.99; 95% CI 0.97, 1.00) (Table 2 and Fig. 2).

**Table 2:** Summary odds ratios (OR) and their 95% confidence interval (CI) estimated by categorical and continuous approaches of dose–response meta-analysis

Group of studies	Number of studies	Medium versus low education level OR (95% CI)	High versus low education level OR (95% CI)	1-year increase in education OR (95% CI)
All studies	94	0.94 (0.83, 1.06)	0.86 (0.72, 1.03)	0.99 (0.97, 1.00)
Study design				
Cohort	2	–	–	0.93 (0.89, 0.97)
Cross-sectional	92	0.95 (0.84, 1.07)	0.88 (0.73, 1.05)	0.99 (0.97, 1.00)
Type of antibiotic misuse				
Use without prescription	70	0.94 (0.82, 1.08)	0.85 (0.68, 1.06)	0.99 (0.97, 1.00)
Storage of antibiotics	10	1.17 (0.93, 1.48)	1.41 (1.22, 1.64)	1.03 (1.01, 1.06)
Non-adherence	9	0.98 (0.65, 1.49)	0.98 (0.71, 1.35)	0.99 (0.96, 1.03)
Several concomitant types of misuse	5	–	–	0.91 (0.87, 0.95)
Country economy				
Low	14	0.84 (0.54, 1.32)	0.69 (0.37, 1.28)	0.97 (0.92, 1.02)
Lower-middle	24	0.94 (0.74, 1.19)	0.70 (0.44, 1.13)	0.97 (0.93, 1.01)
Upper-middle	30	1.02 (0.89, 1.17)	0.95 (0.78, 1.16)	1.00 (0.98, 1.02)
High	25	0.80 (0.66, 0.97)	0.97 (0.74, 1.27)	1.00 (0.97, 1.03)
WHO Region				
Africa	18	0.93 (0.75, 1.16)	0.84 (0.59, 1.21)	0.98 (0.95, 1.01)
Eastern Mediterranean	27	0.91 (0.68, 1.22)	0.64 (0.42, 1.00)	0.96 (0.93, 1.00)
European	18	1.02 (0.81, 1.28)	1.25 (1.00, 1.58)	1.02 (1.00, 1.04)
Region of the Americas	6	1.06 (0.82, 1.38)	0.98 (0.69, 1.38)	1.00 (0.97, 1.03)
South-East Asia	8	0.61 (0.30, 1.23)	0.45 (0.14, 1.41)	0.93 (0.81, 1.06)
Western Pacific	17	0.94 (0.77, 1.14)	1.11 (0.94, 1.31)	1.01 (0.99, 1.03)
Publication year				
Until 2015	42	0.99 (0.81, 1.21)	0.95 (0.74, 1.22)	1.00 (0.97, 1.02)
After 2015	52	0.92 (0.81, 1.05)	0.81 (0.64, 1.02)	0.98 (0.96, 1.00)
Pre-tested or validated questionnaire				
No	26	0.75 (0.59, 0.95)	0.65 (0.41, 1.02)	0.97 (0.94, 1.01)
Yes	68	1.01 (0.89, 1.15)	0.95 (0.80, 1.13)	0.99 (0.98, 1.01)
Restriction, matching or adjustment for age and gender				
No	47	0.86 (0.72, 1.02)	0.70 (0.53, 0.94)	0.97 (0.93, 1.01)
Yes	47	1.00 (0.85, 1.19)	1.05 (0.87, 1.28)	1.00 (0.99, 1.02)
Quality Score				
Lower quality (≤ 3 points)	34	0.84 (0.67, 1.04)	0.76 (0.52, 1.10)	0.98 (0.95, 1.01)
Higher quality (> 3 points)	60	1.00 (0.87, 1.15)	0.93 (0.77, 1.11)	0.99 (0.98, 1.01)

### Subgroup analysis

#### Type of misuse

The categorical and continuous approaches revealed that education level is not associated with unprescribed antibiotics use (OR of 1-year increment = 0.99; 95% CI: 0.97, 1.00) or non-adherence to treatment regimen (OR of 1-year increment = 0.99; 95% CI 0.96, 1.03). Nonetheless, high education level is associated with 41% higher odds of storage of antibiotics (OR = 1.41; 95% CI 1.22, 1.64), compared to low education. The association with antibiotic storage was also observed for medium education level, yet with smaller magnitude (OR = 1.17; 95% CI 0.93, 1.48) (Table 2). These findings are in line with that of the continuous approach in which 1-year increment in education is associated with 3% higher odds of antibiotics storage (OR = 1.03; 95% CI 1.01, 1.06) (Table 2). The association of education with several concomitant types of antibiotic misuse could not be determined using the categorical approach due to insufficient observations, yet the continuous approach showed that each 1-year increase in education is associated with 9% lower odds of antibiotic misuse in general (OR = 0.91; 95% CI 0.87, 0.95) (Table 2).

#### Country economy

In high-wealth countries, the odds of antibiotic misuse are 20% lower in individuals with medium education than in those with low education level (OR = 0.80; 95% CI 0.66, 0.97). In countries with lower-middle economy, high education is associated with 30% reduced odds of antibiotic misuse, compared to low education (OR = 0.70; 95% CI; 0.44, 1.13). Moreover, in these countries, each additional year in education is associated with 3% lower odds of antibiotic misuse (OR = 0.97; 95% CI 0.93, 1.01) (Table 2).

#### WHO regions

In the Eastern Mediterranean region, in reference to individuals with low education level, high education is associated with 36% lower odds of antibiotic misuse (OR = 0.64; 95% CI 0.42, 1.00). In the European region, high education is associated with 25% higher odds of misuse (OR = 1.25; 95% CI 1.00, 1.58). Similarly, in the continuous approach, every additional year in education is associated with 4% lower odds in the Eastern Mediterranean (OR = 0.96; 95% CI 0.93, 1.00) and 2% higher odds in the European regions (OR = 1.02; 95% CI 1.00, 1.04) (Table 2).

#### Publication year

Pooled estimates from studies published after 2015 showed 19% lower odds of antibiotic misuse by individuals with high education level, compared to those with low education level (OR = 0.81; 95% CI 0.64, 1.02). Every additional year in education was associated with 2% reduced odds of antibiotic misuse (OR_after 2015_ = 0.98; 95% CI 0.96, 1.00).

#### Methodological criteria

Sixty eight out of 94 studies applied a previously tested or validated questionnaire. The pooled estimate from those 68 studies did not differ widely from that estimated from all studies for medium education (OR = 1.01; 95% CI 0.89, 1.15) as well as for 1-year increment (OR = 0.99; 95% CI 0.98, 1.01). However, the association of high education with misuse was less strong among studies with validated questionnaire than among all studies taken together (OR = 0.95; 95% CI 0.80, 1.13) (Table 2). Studies that controlled for age and gender, as well as those considered of higher quality did not show any association between education and antibiotic misuse (Table 2).

#### Publication bias

**Fig. 3:**
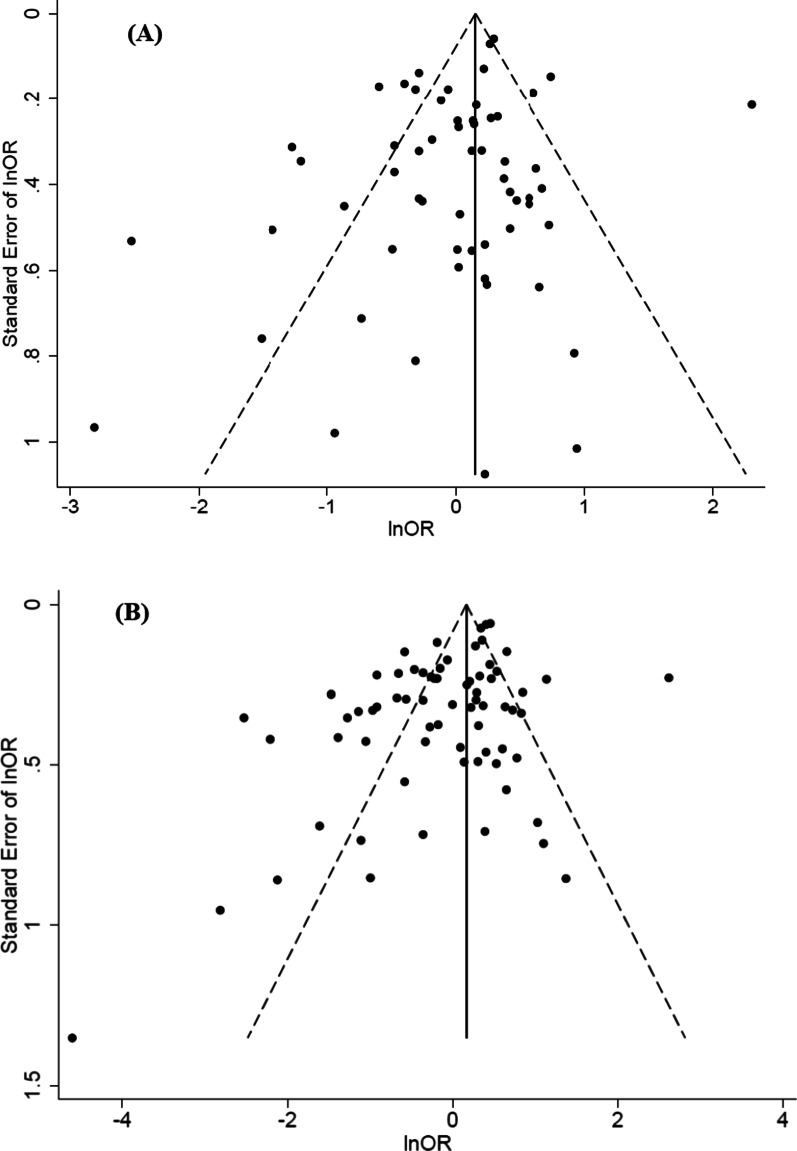
Funnel plot of the studies on education level and antibiotic misuse. **A** Association of medium education with antibiotic misuse. **B** Association of high education with antibiotic misuse

The funnel plot of studies reporting medium education level was slightly skewed to the left (Fig. 3A), but publication bias was neither confirmed by Eggers’s test (*p* value = 0.065), nor by the trim-and-fill analysis that did not suggest the addition of any study. As for those studies that assessed high education level, the funnel plot was also slightly skewed to the left (Fig. 3B). Egger’s test suggested the presence of publication bias (*p* value = 0.001), but the trim-and-fill analysis did not suggest the addition of any study.

## Discussion

We carried out a systematic review and dose–response meta-analysis to summarize the association between education level as a dose and misuse of antibiotics by the general population. Data from studies included in the meta-analysis fit well with a linear association between education and antibiotic misuse. We found no association between a one-year increment in education and the occurrence of antibiotic misuse. In the categorical analysis, we observed that individuals with high education level are, in general, at lower odds of antibiotic misuse than those with low education level. However, these results were not confirmed for the European studies group and the storage-type group of misuse.

The likelihood of using antibiotics without prescription as well as that of non-adherence to treatment is similar for low, medium and highly educated individuals. Notably, the odds of storing antibiotics at home for future need is larger for highly educated people than for those with low education level.

People with low education are more susceptible to comorbidities and thus, are more exposed to medicines than individuals with higher education [24]. Education is strongly associated with socioeconomic status, especially with income [25]. On the one hand, financially disadvantaged people regularly report forgone care [26], and shorten their treatment or buy fewer doses than prescribed, due to cost [27]. In addition, self-medication is most often the only available choice for people with limited financial resources, especially in countries with constrained access to health facilities [28]. On the other hand, individuals with higher socioeconomic status, i.e., higher education, have more social networking which favors their access to unprescribed antibiotics. Moreover, they are more likely to have better economic affordability to buy and store non-reimbursed antibiotics [29]. This could, at least partially, explain our findings concerning a higher misuse likelihood in European countries. Regulations to control the dispensing of antibiotics should be further enforced as more than half of the antibiotics worldwide are still dispensed without prescription [30].

Health literacy significantly contributes to health status and medicines use [31]. Individuals with low education level are characterized by poorer health literacy skills than those with high education [32]. The lack of access to healthcare of less educated people also reduces their health literacy [33]. Nonetheless, limited health literacy is not only restricted to people with low education [33]. In wealthy countries such as in Europe the prevalence of low health literacy ranges between 30 and 60% [34]. Indeed, the health literacy of the population is also influenced by factors other than education level, such as the ease of public understanding to the available health related information [35] as well as the proficiency of the healthcare provider in communicating the information to the patient [33, 36]. Cultural differences and divergence in opinions and beliefs may also influence population´s behaviours towards a specific health issue, including towards the medicines used in it. In the context of antibiotics, it was reported that in certain settings health literacy concerning antibiotic use was insufficient among highly educated people [37]. Insufficient knowledge and misconceptions about antibiotics were also reported both in developed and developing countries [38].

The odds of antibiotic misuse by highly educated people decreased after 2015, which could be related to the global efforts exerted by WHO as well as to the educational campaigns and antibiotic stewardship programs undertaken in many parts of the world to increase the awareness about antibiotic resistance [16, 39]. Highly educated people have better access to internet-based health information than socioeconomically disadvantaged individuals [40], including information on antibiotic use.

This meta-analysis has various strengths. To the best of our knowledge, it is the first to assess the association between education and antibiotic misuse. To allow for comparability across studies, we transformed the education level to years of education, adapting to the setting of each country, before establishing cut-off limits for categories of education levels. We also provided a summary measure of association per increase of 1 year of education. In addition, we presented stratified analysis by country wealth as proxy of socioeconomic status.

Nonetheless, our study suffers from limitations. All except two studies included in the meta-analysis are of cross-sectional design, a design that, theoretically, does not allow for causal inference, due to the fact that exposure and outcome are measured concomitantly. However, education is not a transient factor, but a cumulative characteristic. The fact that exposure and outcome are measured concomitantly does not imply that the level of education may have been acquired after the occurrence of misuse.

High amount of heterogeneity existed across studies. Meta-analysis experts highlight that heterogeneity is expected in any meta-analysis [41], especially in a case of meta-analyses such as ours with large variability in study setting, population characteristics, definition of education level, and period of antibiotic misuse. Therefore, we accounted for heterogeneity by applying random-effect models more adapted to meta-analyses with substantial amounts of heterogeneity.

Furthermore, not all studies provided measures of association adjusted for potential confounders or reported restriction, matching or confounding variables. Studies that controlled for age and gender yielded a summary estimate closer to the null value than studies that did not control for those variables. Likewise, around one-third of studies used non-validated questionnaires. These studies yielded a pooled estimated farther from the null value than with validated instruments.

Finally, although some elements of publication bias were observed in studies that assessed the association of high education with antibiotic misuse, this was unlikely to affect our results as showed by the absence of additional studies in the trim-and-fill analysis.

## Conclusions

This meta-analysis shed the light on the importance of orienting intervention programs to improve the rationale use of antibiotics to all communities independent of their educational level. It also pointed out on the considerable need for cohort studies that examined the association between education and antibiotic misuse and control the measures of association for potentially confounding variables. Measuring the interaction between various socioeconomic indicators such as income and education on antibiotic misuse, would help understand better the socioeconomic properties of antibiotic misuse and thus allow for better control.

## Supplementary Information

**Additional file 1.** Forest plot of studies examining the association between medium education and antibiotic misuse

**Additional file 2.** Forest plot of studies examining the association between high education and antibiotic misuse

**Additional file 3.** Additional reference lists: review reports and studies included in the meta-analysis

## Data Availability

The datasets used and/or analysed during the current study are available from the corresponding author on reasonable request.

## Abbreviations

CI: Confidence interval
OR: Odds ratio
RR: Risk ratio
WHO: World Health Organization
