# Supplementary material for: Education level and misuse of antibiotics in the general population: a systematic review and dose–response meta-analysis
Source: Antimicrob Resist Infect Control. 2022 Feb 3;11:24. doi: 10.1186/s13756-022-01063-5 (PMC8815169; doi:10.1186/s13756-022-01063-5)

Forest plot of studies examining the association between medium education and antibiotic misuse

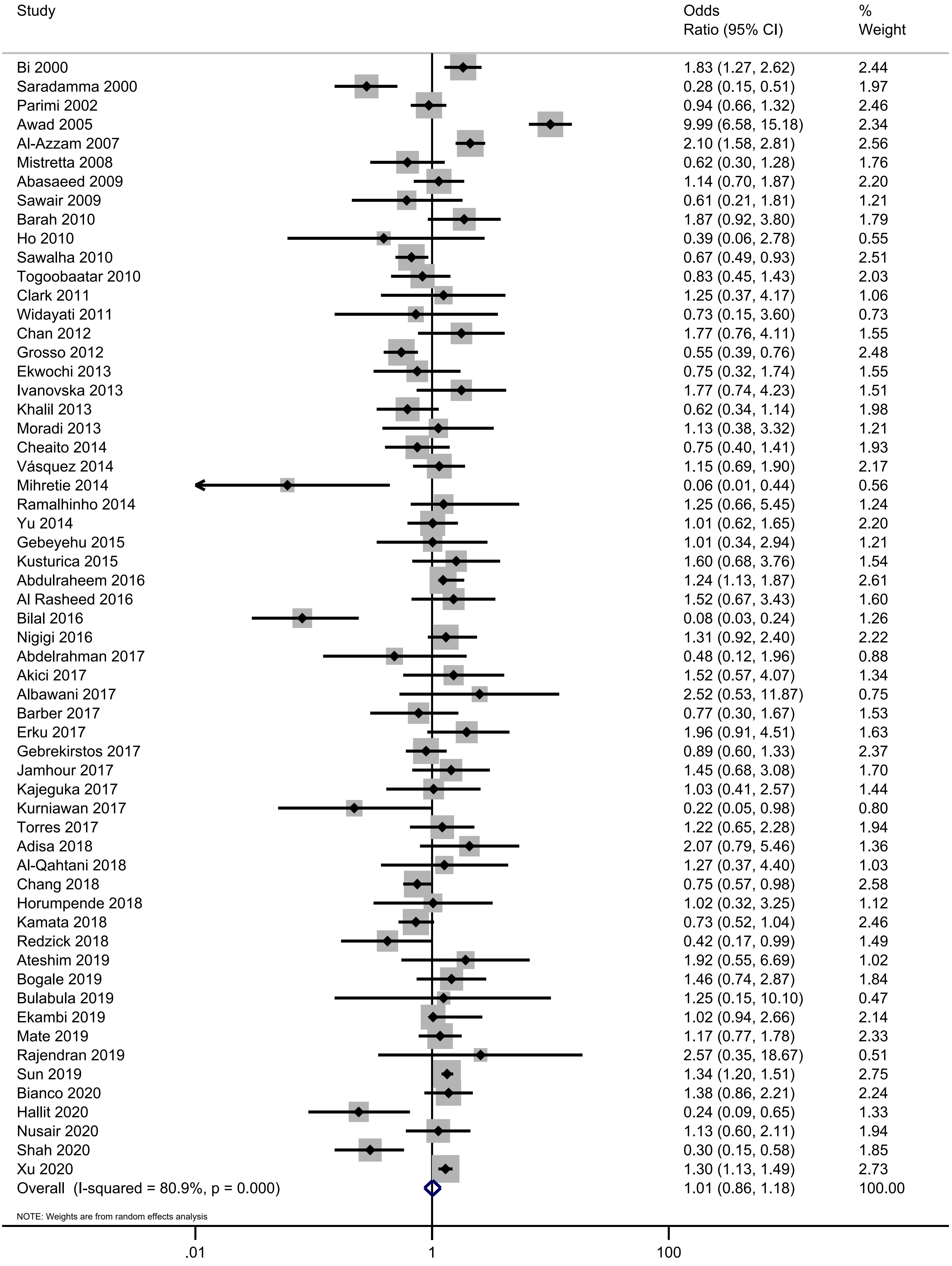

Supplement: Supplementary file 1 — Additional file 1. Forest plot of studies examining the association between medium education and antibiotic misuse [file 13756_2022_1063_MOESM1_ESM.pdf]
