# Supplementary material for: Education level and misuse of antibiotics in the general population: a systematic review and dose–response meta-analysis
Source: Antimicrob Resist Infect Control. 2022 Feb 3;11:24. doi: 10.1186/s13756-022-01063-5 (PMC8815169; doi:10.1186/s13756-022-01063-5)
Supplement: Supplementary file 2 — Additional file 2. Forest plot of studies examining the association between high education and antibiotic misuse [file 13756_2022_1063_MOESM2_ESM.pdf]

Forest plot of studies examining the association between high education and antibiotic misuse

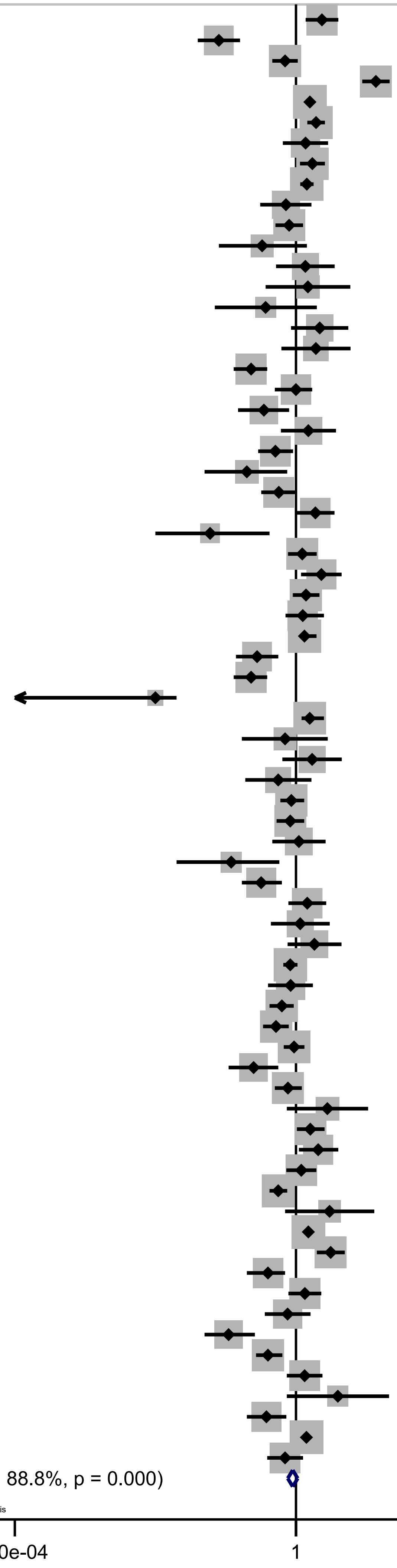

Study	Odds Ratio (95% CI)	% Weight
Bi 2000	2.34 (1.37, 4.01)	1.60
Saradamma 2000	0.08 (0.04, 0.16)	1.43
Parimi 2002	0.70 (0.46, 1.06)	1.72
Awad 2005	13.73 (8.77, 21.51)	1.69
Grigoryan 2006	1.58 (1.41, 1.78)	1.92
Al-Azzam 2007	1.93 (1.45, 2.58)	1.82
Mistretta 2008	1.37 (0.65, 2.86)	1.38
Abasaeed 2009	1.71 (1.14, 2.58)	1.72
Ilhan 2009	1.43 (1.15, 1.78)	1.87
Sawair 2009	0.72 (0.31, 1.66)	1.28
Barah 2010	0.80 (0.51, 1.26)	1.68
Ho 2010	0.33 (0.08, 1.43)	0.77
Landers 2010	1.36 (0.52, 3.55)	1.15
Clark 2011	1.48 (0.37, 5.93)	0.80
Widayati 2011	0.37 (0.07, 1.98)	0.64
Chan 2012	2.18 (0.85, 5.55)	1.18
Elberry 2012	1.92 (0.62, 5.97)	1.00
Abobotain 2013	0.23 (0.13, 0.39)	1.58
Ecker 2013	1.00 (0.50, 1.70)	1.52
Ekwochi 2013	0.35 (0.15, 0.80)	1.28
Ivanovska 2013	1.50 (0.61, 3.71)	1.21
Khalil 2013	0.51 (0.29, 0.91)	1.56
Moradi 2013	0.20 (0.05, 0.75)	0.83
Cheaito 2014	0.57 (0.32, 1.02)	1.55
Hu 2014	1.89 (1.01, 3.53)	1.50
Mihretie 2014	0.06 (0.01, 0.42)	0.54
Ding 2015	1.23 (0.77, 1.97)	1.67
Kusturica 2015	2.30 (1.18, 4.46)	1.46
Pavyde 2015	1.39 (0.90, 2.16)	1.70
Yousif 2015	1.25 (0.71, 2.50)	1.50
Abdulraheem 2016	1.32 (1.18, 1.96)	1.85
Al Rasheed 2016	0.28 (0.14, 0.56)	1.43
Aleem 2016	0.23 (0.13, 0.39)	1.58
Bilal 2016	0.01 (0.00, 0.02)	0.32
Nigigi 2016	1.57 (1.20, 2.50)	1.76
Abdelrahman 2017	0.70 (0.17, 2.83)	0.79
Akici 2017	1.70 (0.64, 4.48)	1.14
Albawani 2017	0.56 (0.19, 1.66)	1.04
Gebrekirstos 2017	0.86 (0.60, 1.31)	1.74
Hassali 2017	0.83 (0.53, 1.31)	1.68
Kajeguka 2017	1.10 (0.46, 2.64)	1.24
Kurniawan 2017	0.12 (0.02, 0.58)	0.63
Senadheera 2017	0.32 (0.17, 0.63)	1.47
Torres 2017	1.45 (0.78, 2.69)	1.51
Adisa 2018	1.15 (0.44, 3.02)	1.15
Al-Qahtani 2018	1.83 (0.76, 4.44)	1.23
Chang 2018	0.83 (0.66, 1.05)	1.86
Cheng 2018	0.84 (0.40, 1.74)	1.39
Dar-Odeh 2018	0.63 (0.42, 0.93)	1.73
El-sherbiny 2018	0.52 (0.34, 0.79)	1.71
Kamata 2018	0.94 (0.67, 1.32)	1.78
Redzick 2018	0.25 (0.11, 0.56)	1.30
Tong 2018	0.77 (0.50, 1.21)	1.69
Ateshim 2019	2.80 (0.74, 10.63)	0.84
Bulabula 2019	1.60 (1.03, 2.55)	1.68
Ekambi 2019	2.07 (1.10, 4.00)	1.48
Mate 2019	1.19 (0.73, 1.95)	1.64
Mukattash 2019	0.56 (0.42, 0.75)	1.82
Rajendran 2019	3.01 (0.70, 13.00)	0.76
Sun 2019	1.50 (1.33, 1.70)	1.91
Voidăzan 2019	3.12 (1.98, 4.94)	1.68
Afari-Asiedu 2020	0.40 (0.20, 0.70)	1.50
Bianco 2020	1.34 (0.78, 2.29)	1.60
Elmahi 2020	0.76 (0.36, 1.61)	1.37
Hallit 2020	0.11 (0.05, 0.26)	1.29
Mallah 2020	0.40 (0.27, 0.64)	1.70
Nusair 2020	1.33 (0.74, 2.38)	1.55
Rathish 2020	3.95 (0.74, 21.10)	0.63
Shah 2020	0.38 (0.20, 0.73)	1.48
Xu 2020	1.41 (1.22, 1.63)	1.91
Moktan 2021	0.70 (0.39, 1.26)	1.55
Overall (I-squared = 88.8%, p = 0.000)	0.90 (0.76, 1.06)	100.00

NOTE: Weights are from random effects analysis
