# Supplementary material for: Education level and misuse of antibiotics in the general population: a systematic review and dose–response meta-analysis
Source: Antimicrob Resist Infect Control. 2022 Feb 3;11:24. doi: 10.1186/s13756-022-01063-5 (PMC8815169; doi:10.1186/s13756-022-01063-5)
Supplement: Supplementary file 3 — Additional file 3. Additional reference lists: review reports and studies included in the meta-analysis [file 13756_2022_1063_MOESM3_ESM.pdf]

Additional file 3.

Reference list of examined review reports

1. Morgan DJ, Okeke IN, Laxminarayan R, et al. Non-prescription antimicrobial use worldwide: a systematic review. Lancet Infect Dis. 2011; https://doi.org/10.1016/S1473-3099(11)70054-8
2. Torres NF, Chibi B, Kuupiel D, et al. The use of non-prescribed antibiotics; prevalence estimates in low-and-middle-income countries. A systematic review and meta-analysis. Arch Public Health. 2021; https://doi.org/10.1186/s13690-020-00517-9
3. Alhomoud F, Aljamea Z, Almahasnah R, et al. Self-medication and self-prescription with antibiotics in the Middle East-do they really happen? A systematic review of the prevalence, possible reasons, and outcomes. Int J Infect Dis. 2017; https://doi.org/10.1016/j.ijid.2017.01.014
4. Torres NF, Chibi B, Middleton LE, et al. Evidence of factors influencing self-medication with antibiotics in low and middle-income countries: a systematic scoping review. Public Health. 2019; https://doi.org/10.1016/j.puhe.2018.11.018
5. De Sanctis V, Soliman AT, Daar S, et al. Prevalence, attitude and practice of self-medication among adolescents and the paradigm of dysmenorrhea self-care management in different countries. Acta Biomed. 2020;91(1):182-92. https://doi.org/10.23750/abm.v91i1.9242
6. Ocan M, Obuku EA, Bwanga F, et al. Household antimicrobial self-medication: a systematic review and meta-analysis of the burden, risk factors and outcomes in developing countries. BMC Public Health. 2015; https://doi.org/10.1186/s12889-015-2109-3
7. Xu R, Mu T, Wang G, et al. Self-medication with antibiotics among university students in LMIC: A systematic review and meta-analysis. J Infect Dev Ctries. 2019; https://doi.org/10.3855/jidc.11359
8. Grigoryan L, Germanos G, Zoorob R, et al. Use of antibiotics without a prescription in the U.S. population: A scoping review. Ann Intern Med. 2019; https://doi.org/10.7326/M19-0505
9. Kardas P, Devine S, Golembesky A, et al. A systematic review and meta-analysis of misuse of antibiotic therapies in the community. Int J Antimicrob Agents. 2005; https://doi.org/10.1016/j.ijantimicag.2005.04.017
10. Gualano MR, Gili R, Scaioli G, et al. General population's knowledge and attitudes about antibiotics: a systematic review and meta-analysis. Pharmacoepidemiol Drug Saf. 2015; https://doi.org/10.1002/pds.3716

Reference list of the 85 studies included in the meta-analysis

1. Afari-Asiedu S, Oppong FB, Tostmann A, et al. Determinants of inappropriate antibiotics use in rural central Ghana using a mixed methods approach. Front Public Health. 2020; https://doi.org/10.3389/fpubh.2020.00090
2. Ho J, Taylor DM, Cabalag MS, et al. Factors that impact on emergency department patient compliance with antibiotic regimens. Emerg Med J. 2010; https://doi.org/10.1136/emj.2009.081984
3. Moktan D, Shehnaz SI. Factors driving self-medication with antimicrobials in Karaikal, Puducherry, India. J Pharmacol Pharmacother. 2021; https://doi.org/10.4103/jpp.JPP_21_20
4. Bianco A, Licata F, Zucco R, et al. Knowledge and practices regarding antibiotics use: Findings from a cross-sectional survey among Italian adults. Evol Med Public Health. 2020; https://doi.org/10.1093/emph/eoaa028
5. Elmahi OKO, Balla SA, Khalil HA. Self-medication with antibiotics and its predictors among the population in Khartoum Locality, Khartoum State, Sudan in 2018. International Journal of Tropical Disease & Health. 2020; https://doi.org/10.9734/IJTDH/2020/v41i430267
6. Hallit S, Zahreddine L, Saleh N, et al. Practice of parents and pharmacists regarding antibiotics use in pediatrics: A 2017 cross-sectional study in Lebanese community pharmacies. J Eval Clin Pract. 2020; https://doi.org/10.1111/jep.13165

7. Mallah N, Badro DA, Figueiras A, et al. Association of knowledge and beliefs with the misuse of antibiotics in parents: A study in Beirut (Lebanon). PLoS One. 2020; https://doi.org/10.1371/journal.pone.0232464
8. Nusair MB, Al-Azzam S, Alhamad H, et al. The prevalence and patterns of self-medication with antibiotics in Jordan: A community-based study. Int J Clin Pract. 2021; https://doi.org/https://doi.org/10.1111/ijcp.13665
9. Rathish D, Wickramasinghe ND. Prevalence, associated factors and reasons for antibiotic self-medication among dwellers in Anuradhapura: a community-based study. Int J Clin Pharm. 2020; https://doi.org/10.1007/s11096-020-01065-6
10. Shah S, Abbas, G., ur Rehman, A., Chanuhdary, Z., Ja Ved, S., Aslam, A., Shah, A.M., Rashid Khan, M., Kazmi, A., Noureen, S.,. Exploring the knowledge and self-prescription with antibiotics in Punjab, Pakistan. Latin American Journal of Pharmacy. 2020;39(5):869-73.
11. Xu Y, Lu J, Sun C, et al. A cross-sectional study of antibiotic misuse among Chinese children in developed and less developed provinces. J Infect Dev Ctries. 2020; https://doi.org/10.3855/jidc.11938
12. Ateshim Y, Bereket B, Major F, et al. Prevalence of self-medication with antibiotics and associated factors in the community of Asmara, Eritrea: a descriptive cross sectional survey. BMC Public Health. 2019; https://doi.org/10.1186/s12889-019-7020-x
13. Bogale AA, Amhare AF, Chang J, et al. Knowledge, attitude, and practice of self-medication with antibiotics among community residents in Addis Ababa, Ethiopia. Expert Rev Anti Infect Ther. 2019; https://doi.org/10.1080/14787210.2019.1620105
14. Bulabula ANH, Dramowski A, Mehtar S. Antibiotic use in pregnancy: knowledge, attitudes and practices among pregnant women in Cape Town, South Africa. J Antimicrob Chemother. 2020; https://doi.org/10.1093/jac/dkz427
15. Elong Ekambi GA, Okalla Ebongue C, Penda IC, et al. Knowledge, practices and attitudes on antibiotics use in Cameroon: Self-medication and prescription survey among children, adolescents and adults in private pharmacies. PLoS One. 2019; https://doi.org/10.1371/journal.pone.0212875
16. Mate I, Come CE, Goncalves MP, et al. Knowledge, attitudes and practices regarding antibiotic use in Maputo City, Mozambique. PLoS One. 2019; https://doi.org/10.1371/journal.pone.0221452
17. Mukattasha TL, Alkhatatbeha MJ, Andrawosa S, et al. Parental self-medication of antibiotics for children in Jordan. JPHSR. 2019; https://doi.org/https://doi.org/10.1111/jphs.12331
18. Rajendran A, Kulirankal KG, Rakesh PS, et al. Prevalence and pattern of antibiotic self-medication practice in an urban population of Kerala, India: A cross-sectional study. Indian J Community Med. 2019; https://doi.org/10.4103/ijcm.IJCM_33_19
19. Sun C, Hu YJ, Wang X, et al. Influence of leftover antibiotics on self-medication with antibiotics for children: a cross-sectional study from three Chinese provinces. BMJ Open. 2019; https://doi.org/10.1136/bmjopen-2019-033679
20. Voidazan S, Moldovan G, Voidazan L, et al. Knowledge, Attitudes And Practices Regarding The Use Of Antibiotics. Study On The General Population Of Mures County, Romania. Infect Drug Resist. 2019; https://doi.org/10.2147/IDR.S214574
21. Adisa R, Orherhe OM, Fakeye TO. Evaluation of antibiotic prescriptions and use in under-five children in Ibadan, SouthWestern Nigeria. Afr Health Sci. 2018; https://doi.org/10.4314/ahs.v18i4.40
22. Al-Qahtani MA, Amin HS, Al-Qahtani AA, et al. Self-medication with Antibiotics in a primary care setting in King Khalid University Hospital, Riyadh, Saudi Arabia. J Family Community Med. 2018; https://doi.org/10.4103/jfcm.JFCM_124_17
23. Chang J, Lv B, Zhu S, et al. Non-prescription use of antibiotics among children in urban China: a cross-sectional survey of knowledge, attitudes, and practices. Expert Rev Anti Infect Ther. 2018; https://doi.org/10.1080/14787210.2018.1425616
24. Cheng J, Coope, C., Chai, J., Oliver, I., Kessel, A., Wang, D, Sun, Y.,. Knowledge and behaviors in relation to antibiotic use among rural residents in Anhui, China. Pharmacoepidemiol Drug Saf 2018; https://doi.org/https://doi.org/10.1002/pds.4429

25. Dar-Odeh SN, Othman, M.B., Bahabri, H.R., Alnazzaw, A.A., Borzangy, S.S., Fadel, T.H., Alolayan, B.A., Abu-Hammad, A.O.,. Antibiotic self-medication for oral conditions: characteristics and associated factor. Pesquisa Brasileira em Odontopediatria e Clinica Integrada. 2018; https://doi.org/http://dx.doi.org/10.4034/PBOCI.2018.181.27
26. El Sherbiny AN, Ibrahim EH, Mohamed M. Assessment of knowledge, attitude and behavior towards antibiotic use in primary health care patients in Fayoum Governorate, Egypt. Alexandaria journal of medicine. 2018; https://doi.org/10.1016/j.ajme.2018.06.001
27. Horumpende PG, Said SH, Mazuguni FS, et al. Prevalence, determinants and knowledge of antibacterial self-medication: A cross sectional study in North-eastern Tanzania. PLoS One. 2018; https://doi.org/10.1371/journal.pone.0206623
28. Kamata K, Tokuda Y, Gu Y, et al. Public knowledge and perception about antimicrobials and antimicrobial resistance in Japan: A national questionnaire survey in 2017. PLoS One. 2018; https://doi.org/10.1371/journal.pone.0207017
29. Ngu RC, Feteh VF, Kika BT, et al. Prevalence and Determinants of Antibiotic Self-Medication among Adult Patients with Respiratory Tract Infections in the Mboppi Baptist Hospital, Douala, Cameroon: A Cross-Sectional Study. Diseases. 2018; https://doi.org/10.3390/diseases6020049
30. Redzic L, Zalihic A. Self-medication with antibiotics in family practice in patients and parents [Samomedikacija antibioticima među pacijentima]. Croatian Journal of Infection [Infektološki glasnik]. 2018;38(3):69-73.
31. Tong S, Pan J, Lu S, et al. Patient compliance with antimicrobial drugs: A Chinese survey. Am J Infect Control. 2018; https://doi.org/10.1016/j.ajic.2018.01.008
32. Abdelrahman TM, Al Saeed MS, Karam RA, et al. Misuse of antibiotics and antibiotic resistance: A public population-based health survey in al Taif- Saudi Arabia. WJPMR. 2017;3(2):54-62.
33. Albawani SM, Hassan YB, Abd-Aziz N, et al. Self-medication with antibiotics in Sana'a City, Yemen. Trop J Pharm Res. 2017; https://doi.org/http://dx.doi.org/10.4314/tjpr.v16i5.30
34. Akici A, Mollahaliloglu S, Donertas B, et al. Patients' attitudes and knowledge about drug use: a survey in Turkish family healthcare centres and state hospitals. Turk J Med Sci. 2017; https://doi.org/10.3906/sag-1608-29
35. Barber DA, Casquejo E, Ybanez PL, et al. Prevalence and correlates of antibiotic sharing in the Philippines: antibiotic misconceptions and community-level access to non-medical sources of antibiotics. Trop Med Int Health. 2017; https://doi.org/10.1111/tmi.12854
36. Erku DA, Mekuria AB, Belachew SA. Inappropriate use of antibiotics among communities of Gondar town, Ethiopia: a threat to the development of antimicrobial resistance. Antimicrob Resist Infect Control. 2017; https://doi.org/10.1186/s13756-017-0272-2
37. Gebrekirstos NH, Workneh BD, Gebregiorgis YS, et al. Non-prescribed antimicrobial use and associated factors among customers in drug retail outlet in Central Zone of Tigray, northern Ethiopia: a cross-sectional study. Antimicrobial Resistance and Infection Control. 2017; https://doi.org/10.1186/s13756-017-0227-7
38. Hassali MA, Arief M, Saleem F, et al. Assessment of attitudes and practices of young Malaysian adults about antibiotics use: a cross-sectional study. Pharm Pract (Granada). 2017; https://doi.org/10.18549/PharmPract.2017.02.929
39. Jamhour A, El-Kheir A, Salameh P, et al. Antibiotic knowledge and self-medication practices in a developing country: A cross-sectional study. Am J Infect Control. 2017; https://doi.org/10.1016/j.ajic.2016.11.026
40. Kajeguka DC, Moses EA. Self-medication practices and predictors for self-medication with antibiotics and antimalarials among community in Mbeya City, Tanzania. Tanzania Journal of Health Research. 2017; http://dx.doi.org/10.4314/thrb.v19i4.6
41. Kurniawan, Posangi J, Rampengan N. Association between public knowledge regarding antibiotics and self-medication with antibiotics in Teling Atas Community Health Center, East Indonesia. Med J Indones. 2017; http://dx.doi.org/10.13181/mji.v26i1.1589
42. Senadheera GP, Sri Ranganathan S, Gunawardane NS, et al. Practice of self-medication with antibiotics in the Colombo district, Sri Lanka. Ceylon Med J. 2017; https://doi.org/10.4038/cmj.v62i1.8439
43. Torres KS, Ochoa A, Encalada D, et al. Prevalence of self-medication with antibiotics in the urban parishes of the city of Cuenca, 2016-2017 [Prevalencia de la automedicacion con

antibioticos en las parroquias urbanas de la ciudad de Cuenca, 2016-2017]. Archivos Venezolanos de Farmacología y Terapéutica. 2017;36(4):130-6.
44. Abdulraheem SI, Adegboye, A., Fatiregun, A.A.,. Self-medication with antibiotics: Empirical evidence from a Nigerian rural population. British Journal of Pharmaceutical Research. 2016; https://doi.org/10.9734/BJPR/2016/25268
45. Aleem MA, Rahman M, Ishfaq M, et al. Determinants of antibiotics misuse by the parents in children: A survey from Northern Region of Saudi Arabia. Bangladesh J Child Health. 2016;40(2):64-71.
46. Al Rasheed A, Yagoub U, Alkhashan H, et al. Prevalence and predictors of self-medication with antibiotics in Al Wazarat Health Center, Riyadh City, KSA. Biomed Res Int. 2016; https://doi.org/10.1155/2016/3916874
47. Bilal M, Haseeb A, Khan MH, et al. Self-medication with antibiotics among people dwelling in rural areas of Sindh. J Clin Diagn Res. 2016; https://doi.org/10.7860/JCDR/2016/18294.7730
48. Nigigi KC. Self medication with antibiotics prior to seeking treatment among adult patients attending outpatient department at Gatundu Sub-county Hospital, Kiambu county, Kenya: Kenyatta University; 2016.
49. Ding L, Sun Q, Sun W, et al. Antibiotic use in rural China: a cross-sectional survey of knowledge, attitudes and self-reported practices among caregivers in Shandong province. BMC Infect Dis. 2015; https://doi.org/10.1186/s12879-015-1323-z
50. Gebeyehu E, Bantie L, Azage M. Inappropriate use of antibiotics and its associated factors among urban and rural communities of Bahir Dar City administration, Northwest Ethiopia. PLoS One. 2015; https://doi.org/10.1371/journal.pone.0138179
51. Kusturica MP, Tomic Z, Bukumiric Z, et al. Antibiotics in serbian households: A source of potential health and environmental threats? Cent Eur J Public Health. 2015; https://doi.org/10.21101/cejph.a4093
52. Pavyde E, Veikutis V, Maciuliene A, et al. Public knowledge, beliefs and behavior on antibiotic use and self-medication in Lithuania. Int J Environ Res Public Health. 2015; https://doi.org/10.3390/ijerph120607002
53. Yousif MA, Abubaker IE. Prevalence, determinants and practices of self-medication with antibiotics – a population based survey in Taif, Kingdom of Saudi Aarabiaksa. Int J Res Pharm Sci. 2015;5(2):51-6.
54. Cheaito L, Azizi S, Saleh N, et al. Assessment of self-medication in population buying antibiotics in pharmacies: a pilot study from Beirut and its suburbs. Int J Public Health. 2014; https://doi.org/10.1007/s00038-013-0493-y
55. Vásquez CSE, Landívar, C.D.J., Astudillo, C.A.J.,. Prevalencia de automedicación con antibióticos y factores asociados, en adultos mayores en el área urbana del cantón cuenca, 2013-2014. Cuenca, Ecuador: University of Cuenca; 2014.
56. Hu J, Wang Z. In-home antibiotic storage among Australian Chinese migrants. Int J Infect Dis. 2014; https://doi.org/10.1016/j.ijid.2014.04.017
57. Mihretie TM. Self-Medication practices with antibiotics among urban dwellers of Bahir Dar Town, North West Ethiopia. Addis Ababa: Addis Ababa University; 2014.
58. Ramalhinho I, Cordeiro C, Cavaco A, et al. Assessing determinants of self-medication with antibiotics among Portuguese people in the Algarve Region. Int J Clin Pharm. 2014; https://doi.org/10.1007/s11096-014-9992-z
59. Yu M, Zhao G, Stalsby Lundborg C, et al. Knowledge, attitudes, and practices of parents in rural China on the use of antibiotics in children: a cross-sectional study. BMC Infect Dis. 2014; https://doi.org/10.1186/1471-2334-14-112
60. Abobotain AH, Sheerah HA, Alotaibi FN, et al. Socio-demographic determinants of antibiotic misuse in children. A survey from the central region of Saudi Arabia. Saudi Med J. 2013;34(8):832-40.
61. Ecker L, Ochoa TJ, Vargas M, et al. Factors affecting caregivers' use of antibiotics available without a prescription in Peru. Pediatrics. 2013; https://doi.org/10.1542/peds.2012-1970
62. Ekwochi U, Chinawa JM, Obi I, et al. Use and/or misuse of antibiotics in management of diarrhea among children in Enugu, Southeast Nigeria. J Trop Pediatr. 2013; https://doi.org/10.1093/tropej/fmt016

63. Ivanovska V, Zdravkovska M, Bosevska G, et al. Antibiotics for upper respiratory infections: public knowledge, beliefs and self-medication in the Republic of Macedonia. Pril (Makedon Akad Nauk Umet Odd Med Nauki). 2013;34(2):59-70.
64. Khalil H, Abdullah, W., Khawaja, N., AlSalem, A., AlHarbi, S., Bin Salleeh, H., Shah, H.A.,. Self-prescribed antibiotics by Saudi patients as a routine self-management of dental problems. Life Science Journal. 2013;10(4):1939-42.
65. Moradi M, Shahraki, H.S., Rezayee, M., Verdi, M.,. Compliance with antimicrobial therapy: Evaluating the related factors. J Pharm Care. 2013;1(2):60-4.
66. Chan YH, Fan MM, Fok CM, et al. Antibiotics nonadherence and knowledge in a community with the world's leading prevalence of antibiotics resistance: Implications for public health intervention. Am J Infect Control. 2012; https://doi.org/10.1016/j.ajic.2011.03.017
67. Elberry AA, Shaik, A., Al-Marzouki, J., Fadul, R. Evaluation of non-prescribed antibiotic use among children with upper respiratory tract infection. Int Res J Pharm Pharmacol. 2012;26:147-52.
68. Grosso G, Marventano S, Ferranti R, et al. Pattern of antibiotic use in the community: nuon-adherence and self-prescription rates in an Italian urban population. Mol Med Rep. 2012; https://doi.org/10.3892/mmr.2012.818
69. Clark DV, Ismayilov A, Bakhishova S, et al. Under-utilization of health care services for infectious diseases syndromes in rural Azerbaijan: a cross-sectional study. BMC Health Serv Res. 2011; https://doi.org/10.1186/1472-6963-11-32
70. Widayati A, Suryawati S, de Crespigny C, et al. Self medication with antibiotics in Yogyakarta City Indonesia: a cross sectional population-based survey. BMC Res Notes. 2011; https://doi.org/10.1186/1756-0500-4-491
71. Barah F, Goncalves V. Antibiotic use and knowledge in the community in Kalamoon, Syrian Arab Republic: a cross-sectional study. East Mediterr Health J. 2010;16(5):516-21.
72. Landers TF, Ferng YH, McLoughlin JW, et al. Antibiotic identification, use, and self-medication for respiratory illnesses among urban Latinos. J Am Acad Nurse Pract. 2010; https://doi.org/10.1111/j.1745-7599.2010.00539.x
73. Sawalha A. Extent of storage and wastage of antibacterial agents in Palestinian households. Pharm World Sci. 2010; https://doi.org/10.1007/s11096-010-9404-y
74. Togoobaatar G, Ikeda N, Ali M, et al. Survey of non-prescribed use of antibiotics for children in an urban community in Mongolia. Bull World Health Organ. 2010; https://doi.org/10.2471/BLT.10.079004
75. Abasaeed A, Vlcek J, Abuelkhair M, et al. Self-medication with antibiotics by the community of Abu Dhabi Emirate, United Arab Emirates. J Infect Dev Ctries. 2009; https://doi.org/10.3855/jidc.466
76. Ilhan MN, Durukan E, Ilhan SO, et al. Self-medication with antibiotics: questionnaire survey among primary care center attendants. Pharmacoepidemiol Drug Saf. 2009; https://doi.org/10.1002/pds.1829
77. Sawair FA, Baqain ZH, Abu Karaky A, et al. Assessment of self-medication of antibiotics in a Jordanian population. Med Princ Pract. 2009; https://doi.org/10.1159/000163041
78. Hadi U, Duerink DO, Lestari ES, et al. Survey of antibiotic use of individuals visiting public healthcare facilities in Indonesia. Int J Infect Dis. 2008; https://doi.org/10.1016/j.ijid.2008.01.002
79. Mistretta A, Grosso G, Contarino F, et al. [Drug misuse in the community: factors which influence patients to self-prescribe antibiotics]. Ann Ig. 2008;20(3):287-95.
80. Al-Azzam SI, Al-Husein BA, Alzoubi F, et al. Self-medication with antibiotics in Jordanian population. Int J Occup Med Environ Health. 2007; https://doi.org/10.2478/v10001-007-0038-9
81. Grigoryan L, Haaijer-Ruskamp FM, Burgerhof JG, et al. Self-medication with antimicrobial drugs in Europe. Emerg Infect Dis. 2006; https://doi.org/10.3201/eid1203.050992
82. Awad A, Eltayeb I, Matowe L, et al. Self-medication with antibiotics and antimalarials in the community of Khartoum State, Sudan. J Pharm Pharm Sci. 2005;8(2):326-31.
83. Parimi N, Pinto Pereira LM, Prabhakar P. The general public's perceptions and use of antimicrobials in Trinidad and Tobago. Rev Panam Salud Publica. 2002; https://doi.org/10.1590/s1020-49892002000700003

84. Bi P, Tong S, Parton KA. Family self-medication and antibiotics abuse for children and juveniles in a Chinese city. Soc Sci Med. 2000; https://doi.org/10.1016/s0277-9536(99)00304-4
85. Saradamma RD, Higginbotham N, Nichter M. Social factors influencing the acquisition of antibiotics without prescription in Kerala State, south India. Soc Sci Med. 2000; https://doi.org/10.1016/s0277-9536(99)00380-9
